# P-1555. Plasma proteomics demonstrate association between IL-6 and expansion of an immunosuppressive monocyte substate during sepsis

**DOI:** 10.1093/ofid/ofaf695.1735

**Published:** 2026-01-11

**Authors:** Pierre Ankomah, Alyssa DuBois, Roby P Bhattacharyya

**Affiliations:** Massachusetts General Hospital, Boston, MA; Broad Institute, Boston, Massachusetts; Massachusetts General Hospital, Boston, MA

## Abstract

**Background:**

The monocyte substate MS1, identified through single-cell RNA sequencing (scRNA-seq), is enriched in sepsis and shares a transcriptional profile with monocytic myeloid-derived suppressor cells (M-MDSCs), which inhibit T cell activation and cytotoxicity in cancer and chronic inflammation. M-MDSCs can be induced by a range of signals, including inflammatory cytokines such as IL-6 and IL-1β, as well as growth factors like GM-CSF and M-CSF involved in emergency myelopoiesis. While MS1 expansion has been observed in sepsis, the cytokine drivers of this response remain incompletely defined.

**Figure 1.**
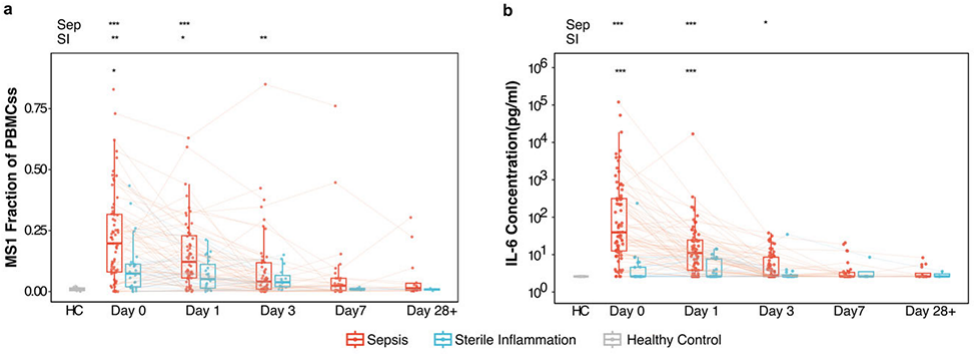
IL-6 levels parallel the early expansion and subsequent decline of an immunosuppressive monocyte state in sepsis. (a) MS1 monocyte fractional abundance within PBMCs decreases progressively from presentation through recovery. (b) IL-6 plasma concentrations follow a similar temporal trajectory. Samples per timepoint: Day 0 – sepsis (n=67), sterile inflammation (n=26); Day 1 – sepsis (n=60), sterile inflammation (n=23); Day 3 – sepsis (n=53), sterile inflammation (n=15); Day 7 – sepsis (n=27), sterile inflammation (n=3); Convalescence (Day 28+) – sepsis (n=14), sterile inflammation (n=3). Points and lines represent individual subjects, colored by phenotype; boxes show median and interquartile range. IL-6 concentrations (pg/mL) were measured in matched plasma samples using multiplex proteomics. Significance was assessed using Wilcoxon rank-sum test with Benjamini-Hochberg correction. Statistical comparisons between sepsis and sterile inflammation (SI) are shown within the plotting area; comparisons versus healthy controls (HC) are shown outside the plotting area. *p < 0.05, **p < 0.01, ***p < 0.001.

**Methods:**

We performed scRNA-seq with paired surface protein profiling on peripheral blood mononuclear cells (PBMCs) from patients with sepsis and sterile inflammation, sampled at presentation to the emergency department and longitudinally through recovery. In total, we analyzed samples from 130 individuals: 71 with sepsis, 27 with sterile inflammation, and 12 healthy controls recruited outside the ED. After quality control, 560,867 high-quality single cells were analyzed using multimodal integration. Matched plasma samples were profiled using a high-plex cytokine proteomics platform to assess candidate inducers of the MS1 state.

**Results:**

MS1 monocytes were elevated at sepsis onset and declined progressively through day 7, remaining low into convalescence (Fig. 1a). Among candidate cytokines, only IL-6 exhibited a parallel temporal decline (Fig. 1b). Spearman correlations between IL-6 levels and MS1 abundance ranged from ρ=0.39 at day 0 to ρ=0.69 at convalescence. Other cytokines, including IL-1β, IL-8, TNF-α, and IL-10, showed less consistent trajectories and weaker correlations.

**Conclusion:**

These findings demonstrate an association between IL-6 and early expansion of an immunosuppressive monocyte state during sepsis, suggesting a putative IL-6–dependent emergency myelopoiesis program. The observed temporal relationship may reflect an early, IL-6–driven adaptation that transitions toward immune restoration during recovery. Defining cytokine–cell state relationships in acute infection may inform strategies to modulate myeloid cell dysregulation in patients with sepsis.

**Disclosures:**

All Authors: No reported disclosures

